# Fractional amplitude of low-frequency fluctuations (fALFF) changes in children with anisometropic amblyopia: A resting-state fMRI study

**DOI:** 10.1097/MD.0000000000051299

**Published:** 2026-10-23

**Authors:** Xueliang Yu, Lisheng Zhao, Weizhao Lu, Di Chen, Dandan Li, Xiujuan Li, Shengdong Nie, Yi Wang, Baojian Wang

**Affiliations:** a Department of Ophthalmology, Dezhou Municipal Hospital, Dezhou, Shandong, China; b Department of Radiology, Dezhou Municipal Hospital, Dezhou, Shandong, China; c School of Radiology, Shandong First Medical University & Shandong Academy of Medical Sciences, Taian, Shandong, China; d Department of Ophthalmology, The Second Affiliated Hospital of Shandong First Medical University, Taian, Shandong, China; e Department of Radiology, The Affiliated Taian City Central Hospital of Qingdao University, Taian, Shandong, China; f School of Health Science and Engineering, University of Shanghai for Science and Technology, Shanghai, China; g Department of PET-CT, The Affiliated Taian City Central Hospital of Qingdao University, Taian, Shandong, China.

**Keywords:** amplitude of low-frequency fluctuation, anisometropic amblyopia, fractional amplitude of low-frequency fluctuation (fALFF), functional magnetic resonance imaging

## Abstract

This study aimed to explore changes in spontaneous brain activity in children with anisometropic amblyopia (CAA) using the fractional amplitude of low-frequency fluctuation (fALFF) analysis method and to determine whether anisometropic amblyopia is associated with changes in brain function. Resting-state functional magnetic resonance imaging data were collected from 16 children with anisometropic amblyopia (CAA group) and 10 healthy children (HC group). fALFF was used to assess spontaneous brain activity. Group differences in fALFF were analyzed using a two-sample *t* test, with age, sex, and head motion included as covariates. The statistical threshold was set at a voxel-level *P* < .001 (uncorrected), combined with a cluster-level family-wise error correction at *P* < .05. Partial Pearson correlation analyses, controlling for age and sex, were used to examine the relationships between fALFF values in regions showing significant group differences and clinical measures (best-corrected visual acuity of the amblyopic eye and magnitude of anisometropia) in the CAA group. Given the exploratory nature of the study, no correction for multiple comparisons was applied. Compared with the HC group, the CAA group showed higher fALFF values in the right middle occipital gyrus and left paracentral lobule. The fALFF value in the left middle frontal gyrus was lower in the CAA group than in the HC group. There were no significant correlations between clinical information and mean fALFF values in the CAA group. This study suggests that CAA may exhibit aberrant spontaneous brain activity within visual and frontal networks, as measured by fALFF. The absence of a direct correlation with standard clinical measures suggests that fALFF may capture distinct, network-level neural alterations in amblyopia. However, whether fALFF can serve as a reliable neuroimaging biomarker remains hypothetical and requires confirmation in larger, well-powered studies with comprehensive visual-function assessments.

## 1. Introduction

Amblyopia is a neurodevelopmental disorder characterized by reduced best-corrected visual acuity (BCVA) in one or both eyes, below the age-appropriate normal limit, due to abnormal visual experience such as form deprivation or binocular interaction during critical periods of visual development.^[1]^ Patients primarily present with impaired visual acuity but often exhibit additional deficits in contrast sensitivity, stereopsis, eye movements, visual attention, and hand-eye coordination. It is widely accepted that amblyopia involves aberrant neural plasticity within the visual cortex.^[2]^ With a global prevalence of approximately 1.44%, amblyopia is a significant public health concern, projected to affect 175 million individuals worldwide by 2030.^[3]^

Resting-state functional magnetic resonance imaging (rs-fMRI) provides a noninvasive tool for investigating brain function. In the resting state, the brain – representing only 2% of body weight – consumes over 20% of the body’s metabolic energy, indicating substantial intrinsic functional activity.^[4]^ Since the introduction of blood oxygen level-dependent (BOLD) fMRI to visual research,^[5]^ this technique has proven valuable for localizing and quantifying visual system function, integrating neural anatomy with physiology.^[6]^ Studies have consistently demonstrated organized low-frequency oscillations in the brain, with high consistency observed in bilateral visual regions, underscoring their physiological relevance to visual processing.^[7,8]^ For instance, Tang et al reported altered amplitudes of low-frequency fluctuation (ALFF) in multiple brain regions, including the temporal lobe, parietal lobule, and frontal gyrus, in patients with anisometropic amblyopia.^[9]^ The ALFF metric, proposed by Zang et al, quantifies the magnitude of these low-frequency oscillations (0.01–0.08 Hz) at the voxel level, reflecting the intensity of regional spontaneous neural activity.^[10]^

Anisometropic amblyopia is a common form of amblyopia, resulting from a significant interocular refractive difference and associated with diverse visual functional impairments.^[11]^ While studies have identified functional alterations in early visual areas associated with the core acuity deficit in amblyopia,^[12]^ a critical unresolved question is which specific brain regions beyond the primary visual cortex exhibit functional alterations underlying these diverse deficits and how such neural abnormalities correlate with clinical visual measures.

To this end, we turned to fractional ALFF (fALFF), an improved variant of ALFF. fALFF is defined as the ratio of power within the low-frequency range to that across the entire detectable frequency spectrum. This normalization confers a key advantage: reduced susceptibility to physiological noise, thereby potentially offering a more specific measure of spontaneous neuronal activity than ALFF.^[13,14]^ Capitalizing on this enhanced specificity, we employed fALFF in children with anisometropic amblyopia (CAA). We hypothesized that fALFF would provide a more specific measure of the spontaneous neural activity alterations associated with this condition. Previous ALFF studies have reported inconsistent results in amblyopia, and the specific alterations in fALFF – particularly in pediatric anisometropic amblyopia – remain largely unexplored.

This study aimed to investigate fALFF differences between CAA and healthy controls and to explore their associations with clinical measures.

## 2. Materials and methods

### 2.1. Subjects

This study comprised 16 children with anisometropic amblyopia (CAA group) and 10 healthy children (HC group) recruited from the ophthalmology clinic of Dezhou Municipal Hospital between November 2021 and May 2023. The inclusion criteria for the CAA group were a diagnosis of anisometropic amblyopia, defined as an interocular spherical equivalent refractive error difference ≥1.00 diopter (D), with BCVA in the amblyopic eye below the age-appropriate normal limit (e.g., >0.2 logMAR for children aged 3 to 5 years and >0.1 logMAR for children aged ≥6 years) after optimal optical correction; right-handedness; age between 3 and 12 years; and no other cause for reduced vision. The inclusion criteria for the HC group were right-handed children aged 3 to 12 years with unaided or corrected visual acuity of 0.0 logMAR (Snellen 1.0 or better) in both eyes and no significant refractive error (spherical equivalent between −0.50 D and +1.00 D). The exclusion criteria for both groups were as follows: any significant medical or psychiatric condition that may interfere with study participation; a history of neurological disorders or serious eye diseases (e.g., strabismus, cataract, glaucoma, and retinal pathologies); any contraindication to MRI scanning (e.g., claustrophobia and presence of ferromagnetic implants); inability to maintain stillness during the MRI procedure, leading to excessive head movement; and any ongoing treatment for amblyopia (e.g., patching and atropine penalization) that might affect the study outcomes. Patients in the CAA group were required to have worn their full optical correction for at least 1 month prior to the MRI scan to ensure stable visual input.

All subjects underwent detailed ophthalmic examinations, including slit-lamp, eye muscle, and fundus examinations, and no abnormalities were found. Refractive errors were measured under cycloplegia (1% cyclopentolate) in all participants. Patients reported no history of serious eye or nervous system diseases. Two children in the HC group were excluded due to excessive head movement during the fMRI scan (see the Pretreatment section for the criteria). Finally, 16 children in the CAA group and 10 children in the HC group were included in the analysis.

The clinical characteristics of the CAA group, including laterality of the amblyopic eye (right:left = 5:11), spherical equivalent refractive error of the amblyopic eye (mean ± standard deviation: +4.25 ± 2.18 D), and the magnitude of anisometropia (mean interocular difference: +2.81 ± 1.05 D), are summarized in Table 1.

**Table 1:** Demographic and clinical characteristics of the study participants.

Characteristic	CAA group (n = 16)	HC group (n = 10)	Statistical test	*P* value
Age (yr), mean ± SD	7.53 ± 2.45	8.70 ± 2.54	Independent *t* test	.254
Sex (male/female)	9/7	6/4	χ^2^	.851
Handedness (right/left)	16/0	10/0	–	–
Amblyopic eye (right/left)	5/11	**–**	–	–
BCVA (logMAR), mean ± SD	0.531 ± 0.296	−0.01 ± 0.057	Independent *t* test	<.001
Spherical equivalent of amblyopic eye (D), mean ± SD	+4.25 ± 2.18	–	–	–
Magnitude of anisometropia (D), mean ± SD	+2.81 ± 1.05	–	–	–
Mean framewise displacement (FD), mean ± SD	0.24 ± 0.12	0.20 ± 0.10	Independent *t* test	.36

Continuous variables (age, BCVA, spherical equivalent, magnitude of anisometropia, and mean FD) are presented as mean ± standard deviation and were compared using independent-samples *t* tests. Categorical variables (sex, handedness, and amblyopic eye) are presented as counts and were compared using the χ^2^ test where applicable.

BCVA = best-corrected visual acuity, CAA = children with anisometropic amblyopia, D = diopters, HC = healthy children, SD = standard deviation.

### 2.2. Data collection

All subjects underwent MRI scans using the GE OPTIMA MR360 1.5T MRI system. The instrument completed the stability tests prior to use. First, the subjects had routine T_1_-weighted brain MRI scans. Following on-site confirmation by the 2 radiologists with more than 5 years of clinical experience that no organic craniocerebral abnormalities were present, structural MRI images were acquired using an inversion-recovery fast spin-echo (IR-FSE) sequence in a sagittal orientation. The scan parameters were as follows: repetition time (TR)/echo time (TE) = 1750/20.430 ms, inversion time (TI) = 720 ms, field of view = 240 mm × 240 mm, matrix = 256 × 256, slice thickness = 1 mm, slice gap = 0 mm, number of signal averages = 1, flip angle = 90°, and 176 sagittal slices covering the whole brain. The parameters were determined based on the manufacturer’s default protocol for pediatric brain imaging at 1.5T.

Before the resting-state BOLD scan, the subjects were instructed to close their eyes, breathe calmly, remain fully conscious, and not engage in any specific thinking activity. To acquire fMRI data, an echo-planar imaging sequence with the following parameters was used: repetition time/echo time = 3000/40 ms, field of view = 240 × 240 mm, matrix = 64 × 64, number of slices = 31, slice thickness = 5 mm, layer spacing = 0 mm, flip angle = 90°, and scan duration was 384 seconds (128 time points). Participants were monitored via a camera and instructed to remain awake with eyes closed; no overt drowsiness was observed.

### 2.3. Data processing and statistical analysis

#### 2.3.1. Pretreatment

The resting-state fMRI data were exported to a GE OPTIMA MR360 1.5T (GE HealthCare) MRI parallel workstation via an optical disk and uploaded to the fMRI-dedicated data processing workstation (Windows system; Matrix Laboratory [MATLAB] 2013b platform; The MathWorks, Inc.). The Data Processing & Analysis for Brain Imaging (DPABI) toolkit (version 6.1, https://rfmri.org/dpabi), based on Statistical Parametric Mapping 8 (SPM8; Wellcome Trust Centre for Neuroimaging, University College London) software, was used to preprocess fMRI data following the standardized rs-fMRI data preprocessing process. The main steps were as follows: image format transformation: the original fMRI and 3-dimensional (3D) T_1-_weighted structural images in DICOM format for each subject were transformed into corresponding images in NIFTI format to apply to the SPM software; since the subjects were not used to the noise at the beginning, the magnetic resonance (MR) signal was unstable, therefore, the subjects’ first 10-time point signals were removed, and the remaining 118-time point signals were further analyzed; slice timing was carried out to correct signals collected at different time points to the same time point; head motion correction was carried out by a linear regression process, moreover, subjects with maximum translation >2 mm or maximum rotation >2° were excluded, 2 HCs were excluded based on this criterion, framewise displacement (FD) was calculated for each subject, and the mean FD did not differ significantly between the CAA group (mean FD = 0.24 ± 0.12 mm) and the HC group (mean FD = 0.20 ± 0.10 mm; *P* = .36); registration was conducted, and the original space of the subjects was registered to the standard space, with the help of T_1_WI structural images, all functional images were registered to the standard space of the Montreal Neurological Institute, and each voxel was resampled to 3 × 3 × 3 mm^3^; image smoothing: using a Gaussian smoothing kernel with a full width at half maximum height (FWHM) of 6 × 6 × 6 mm^3^ to perform spatial 3D Gaussian smoothing on each subject’s fMRI image; linear detrend of the time course was performed; and nuisance covariate regression: the Friston 24-parameter model (including 6 head motion parameters, their previous time points, and squared terms) was used to regress out head motion effects, and signals from white matter and cerebrospinal fluid were also regressed out.

#### 2.3.2. The fALFF calculation

fALFF was calculated using the DPABI toolkit. For each voxel, the preprocessed time series was transformed to the frequency domain via Fast Fourier Transform to obtain the power spectrum. The fALFF value was defined as the ratio of the power within the low-frequency range (0.01–0.08 Hz) to the power across the entire detectable frequency range (0–1/[2 × TR] Hz, i.e., 0–0.167 Hz for TR = 3000 ms). These fALFF maps were then standardized by dividing each voxel’s value by the global mean fALFF value of the whole brain for each subject, producing normalized fALFF maps for group-level analysis.

#### 2.3.3. Statistical analysis

No formal sample size calculation was performed prior to recruitment, given the exploratory nature of this study. Statistical analysis software SPSS Statistics 22.0 (IBM Corporation) was used to analyze demographic and clinical information between the CAA and HC groups. Age and BCVA (BCVA values expressed as logMAR vision) were assessed by two-sample *t* tests, and gender was evaluated by the χ^2^ test. Moreover, a *P* value < .05 was considered statistically significant.

The statistics module in the DPABI toolkit based on SPM8 software was used for statistical analysis. SPM8 was used for consistency with our previous studies and the DPABI version (6.1) employed at the time of analysis. Specifically, two-sample *t* tests were utilized to detect significant statistical differences in the fALFF map between the CAA and HC groups. To control for multiple comparisons and minimize false positives, the statistical threshold was set at a voxel-level *P* < .001 (uncorrected), combined with a cluster-level family-wise error (FWE) correction at *P* < .05, using Gaussian Random Field theory. Additionally, to account for potential confounding factors, mean FD, age, and sex were included as covariates in the two-sample *t* test model. There was no significant difference in the mean FD between the CAA group (mean FD = 0.24 ± 0.12 mm) and the HC group (mean FD = 0.20 ± 0.10 mm; *P* = .36).

Partial Pearson correlation analyses, controlling for age and sex, were used to explore the relationships between the mean fALFF values in significant clusters and clinical variables (BCVA of the amblyopic eye and magnitude of anisometropia) in the CAA group. Given the exploratory nature and small sample size, results were interpreted with caution, and no correction for multiple comparisons was applied. Effect sizes (Pearson’s *r*) with 95% confidence intervals are reported.

## 3. Results

### 3.1. Demographic and clinical information

Table 1 lists the demographic and clinical information of the CAA and HC groups. The 2 groups were well-matched in terms of age (*P* = .254) and gender (*P* = .851). There were significant differences in BCVA (*P* < .001) between the 2 groups.

### 3.2. fALFF results

The primary outcome of this study was the fALFF comparison between the CAA and HC groups. Compared with the HC group, the CAA group showed increased fALFF values in the right middle occipital gyrus (BA19) and left central paracentric lobule (BA6) and decreased fALFF values in the left middle frontal gyrus (BA10), as shown in Table 2 and Figure 1. These results survived correction for multiple comparisons at the cluster level (FWE-corrected *P* < .05). Effect sizes for these between-group differences were large: Cohen’s *d* = 1.76 for the right middle occipital gyrus, Cohen’s *d* = −1.67 for the left middle frontal gyrus, and Cohen’s *d* = 1.96 for the left central paracentric lobule. These effect sizes were calculated for clusters that were selected based on the same group comparison, and therefore may be inflated; they should be interpreted as descriptive rather than unbiased estimates of effect magnitude.

**Table 2:** The brain areas with increased and decreased fALFF values in the CAA group compared with the HC group.

Conditions	Brain region	BA	Cluster volume (mm^3^)	MNI coordinates			*t* value
				X	Y	Z	
CAA > HC	Occipital_Mid_R	19	1405	33	−69	3	4.3621
CAA < HC	Frontal_Mid_L	10	2078	−39	39	21	−4.1441
CAA > HC	Paracentral_Lob_L	6	1539	−3	−27	54	4.8692

BA: Brodmann partition system; MNI coordinates: standard coordinates of the Montreal Neurological Institute (MNI).

CAA = children with anisometropic amblyopia, fALFF = fractional amplitude of low-frequency fluctuation, HC = healthy children.

**Figure 1.**
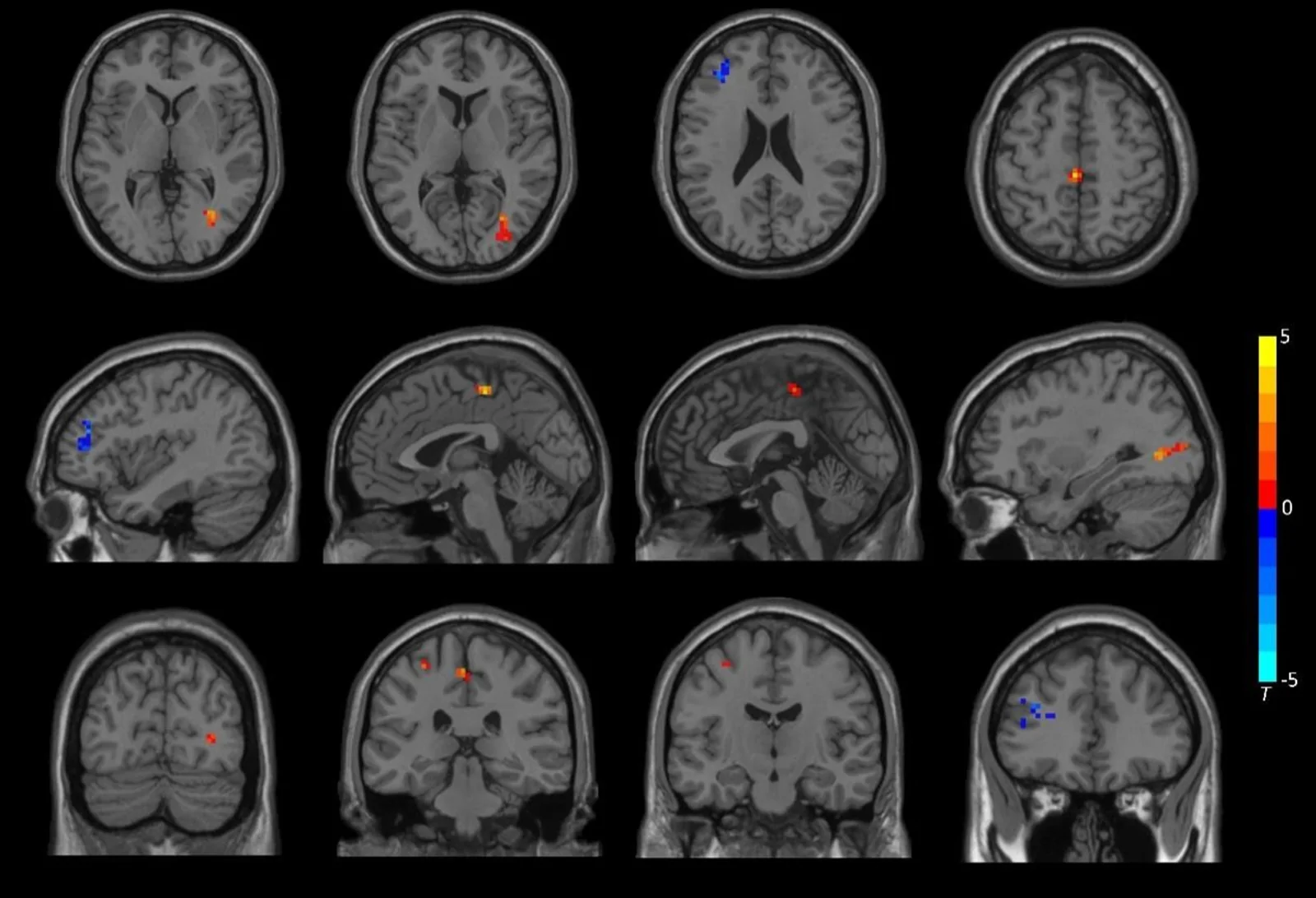
fALFF differences between the CAA and HC groups. The statistical threshold was set at a voxel-level *P* < .001 (uncorrected) and cluster-level FWE correction at *P* < .05. Positive *t* values (warm colors) indicate higher fALFF in CAA versus HC; negative *t* values (cool colors) indicate lower fALFF in CAA versus HC. CAA = children with anisometropic amblyopia, fALFF = fractional amplitude of low-frequency fluctuation, FWE = family-wise error, HC = healthy children.

### 3.3. Correlation analysis

Partial Pearson correlation analyses, controlling for age and sex, were performed to examine the relationships between the mean fALFF values in the identified clusters and the clinical measures (BCVA of the amblyopic eye and magnitude of anisometropia) in the CAA group. No significant correlations were observed after controlling for age and sex (all *P* > .05). The correlation coefficients were small to moderate (*r* ranging from −0.28 to 0.22), and all 95% confidence intervals included 0 (Table 3). Given the exploratory nature of this study, no correction for multiple comparisons was applied to these correlation analyses.

**Table 3:** Correlation analyses between fALFF and clinical variables in the CAA group.

Brain region (cluster)	Clinical variable	Pearson’s *r*	95% confidence interval	*P* value*
Right middle occipital gyrus	BCVA (amblyopic eye)	−0.15	−0.60, 0.38	.58
Right middle occipital gyrus	Magnitude of anisometropia	0.08	−0.43, 0.55	.77
Left central paracentral lobule	BCVA (amblyopic eye)	0.22	−0.31, 0.65	.40
Left central paracentral lobule	Magnitude of anisometropia	−0.28	−0.69, 0.25	.28
Left middle frontal gyrus	BCVA (amblyopic eye)	−0.10	−0.57, 0.42	.70
Left middle frontal gyrus	Magnitude of anisometropia	0.05	−0.45, 0.53	.85

BCVA = best-corrected visual acuity, CAA = children with anisometropic amblyopia, fALFF = fractional amplitude of low-frequency fluctuation.

* *P* values are derived from partial correlation analyses controlling for age and sex.

## 4. Discussion

fALFF is an rs-fMRI analysis technique used to measure spontaneous fluctuations in BOLD-fMRI signal intensity associated with neural activity, reflecting the intensity of local spontaneous brain activity at rest. Compared with ALFF, fALFF’s normalization across the frequency spectrum reduces sensitivity to physiological noise, potentially offering a more specific measure of neuronal activity. The rs-fMRI technique based on fALFF has been widely used in studying various ocular diseases, helping to understand the relationship between ophthalmic disease characteristics and changes in local brain functional areas. fALFF has also been successfully applied to children with strabismic amblyopia, revealing spontaneous activity alterations in frontal and parietal regions.^[15]^

The known functions of the occipital lobe are mainly related to visual information processing; the visual cortex (Brodmann area [BA] 17/18/19) is located here and serves as the starting point and center of the cerebral cortex for visual nerve processing. Our results showed increased fALFF in the right middle occipital gyrus (BA19) in the CAA group, suggesting increased spontaneous activity in the visual cortex of CAA. Previous studies have shown inconsistent results. Dai et al reported that ALFF in the bilateral frontal, temporal, and occipital lobes was lower in the amblyopia group than in HCs.^[16]^ However, Liang et al reported that amblyopic children mainly exhibited increased ALFF in parts of the bilateral calcarine (BA17), the left middle occipital gyrus (BA18/19), and the left postcentral gyrus (BA2), whereas amblyopic adults showed decreased ALFF in the bilateral precuneus cortex (part of BA7).^[11]^

The increased fALFF in the middle occipital gyrus (BA19) in this study may be related to impaired visual input from the macula lutea. Anisometropia makes it difficult to form clear images on the macula lutea of the amblyopic eye, leading to impaired visual information.^[17]^ BA19 receives indirect visual input via earlier visual areas (V1 and V2), and the increased fALFF observed here may reflect compensatory recruitment of higher-order visual areas to maintain visual function and progressively transmit impaired information. Another explanation is based on neural redundancy. Many studies have confirmed that amblyopia is a central nervous system developmental disorder caused by abnormal visual experiences during visual development.^[18,19]^ Normal central nervous system development involves synaptic pruning and apoptosis of redundant neurons.^[20]^ Abnormal visual experiences, such as anisometropia during development, may inhibit normal nervous system development,^[21]^ resulting in the persistence of excessive, functionally less efficient neurons in visual-related areas, ultimately leading to increased spontaneous brain activity. Similarly, a recent fALFF study in children with deprivation amblyopia also reported elevated spontaneous activity in higher-order visual areas.^[22]^

Furthermore, we observed increased fALFF in the left central paracentral lobule in the CAA group. Due to refractive aberration, the visual information received by amblyopic children is defective,^[23]^ and their stereopsis is impaired. The increased fALFF in this motor-related region may reflect compensatory visuomotor integration, consistent with prior reports of altered spontaneous neural activity within visual-motor pathways in visual impairment.^[24]^ However, this interpretation remains speculative, and further studies are needed to clarify the functional role of the paracentral lobule in amblyopia.

Consistent with prior research, we found decreased fALFF in the left middle frontal gyrus in the CAA group.^[16]^ The frontal gyrus is associated with high-level executive functions, including the “what” and “where” visual pathways.^[25]^ Hirsch et al reported^[26]^ that the left medial frontal gyrus is the only cortical region in which 3 neurocognitive systems (same-different discrimination, object naming, and integer computation) are shared. The decreased fALFF in this area suggests reduced spontaneous brain activity, possibly attributable to diminished visual input and associated top-down processing demands in anisometropic amblyopia.

Beyond the well-documented eye movement deficits in amblyopia (e.g., fixation stability, saccades, and smooth pursuit),^[27]^ our study reveals a more complex structure–function relationship. We found no significant correlation between fALFF values in the identified clusters and standard clinical measures (BCVA or anisometropia magnitude) after controlling for age and sex. The absence of significant correlations may reflect limited statistical power rather than a true absence of relationships. The pathophysiology of amblyopia is dynamic, influenced by multiple factors such as age of onset, disease duration, and individual compensatory mechanisms. Any interpretation that fALFF captures distinct neural-level information remains hypothetical in this small exploratory sample.

### 4.1. Limitations and future directions

This study has several limitations. First, the relatively small sample size limits statistical power to detect subtle effects and precludes generalization of our findings. Additionally, this was a single-center study, which may limit the generalizability of the findings. Second, although we mitigated physiological noise using fALFF and nuisance regression, the BOLD signal remains susceptible to confounds from breathing and heartbeat. Third, the cross-sectional design precludes causal inferences regarding the relationship between altered fALFF and amblyopia progression. Fourth, clinically relevant visual-function measures, such as stereoacuity and fixation stability, were not available for correlation analyses. Fifth, we did not find strong evidence for lateralization specific to amblyopic eye laterality in this cohort; this should be explicitly investigated in future studies with larger samples balanced for eye dominance. Additionally, while we employed cluster-level FWE correction, future studies could benefit from more stringent thresholds or nonparametric methods.

To address these limitations, future work will prioritize expanding the sample size. This will allow for a more comprehensive evaluation of the relationship between amblyopia and fALFF and enable correlation analyses with a wider range of clinical data (e.g., stereoacuity and fixation stability). These steps will enhance the robustness of the findings and provide valuable insights for modeling amblyopia.

Additionally, it is worth noting that the structural MRI sequence used in this study was an IR-FSE sequence (TR/TE/TI = 1750/20.43/720 ms, flip angle = 90°). This sequence differs fundamentally from the conventional T1-weighted 3D gradient-echo sequences (e.g., MPRAGE or FSPGR) that are typically recommended for tissue segmentation and spatial normalization in fMRI studies. Although the parameters followed the manufacturer’s default pediatric protocol at 1.5T, the IR-FSE sequence with a relatively long TR and TE provides a mixed T1/PD/T2 contrast rather than pure T1 weighting. This may have affected the accuracy of gray matter/white matter segmentation and registration to standard templates. Future studies should consider acquiring a dedicated high-resolution 3D T1-weighted gradient-echo sequence (e.g., BRAVO or FSPGR with short TR/TE and a low flip angle) for better structural preprocessing.

## 5. Conclusion

In conclusion, this exploratory rs-fMRI study suggests that CAA may exhibit altered spontaneous brain activity in visual and frontal regions as measured by fALFF. These neural changes were not significantly correlated with visual acuity or anisometropia magnitude in this small sample. While these findings may help characterize network-level alterations associated with amblyopia, larger studies with richer visual-function measures and longitudinal designs are needed before fALFF can be considered a reliable neuroimaging biomarker.

## Author contributions

**Conceptualization:** Xiujuan Li, Yi Wang.

**Investigation:** Baojian Wang.

**Methodology:** Lisheng Zhao, Di Chen, Dandan Li.

**Project administration:** Shengdong Nie.

**Resources:** Xiujuan Li, Yi Wang.

**Software:** Weizhao Lu.

**Supervision:** Di Chen, Shengdong Nie, Yi Wang.

**Visualization:** Baojian Wang.

**Writing – original draft:** Xueliang Yu.

**Writing – review & editing:** Baojian Wang.
